# Detection of Human Cholangiocarcinoma Markers in Serum Using Infrared Spectroscopy

**DOI:** 10.3390/cancers13205109

**Published:** 2021-10-12

**Authors:** Patutong Chatchawal, Molin Wongwattanakul, Patcharaporn Tippayawat, Kamilla Kochan, Nichada Jearanaikoon, Bayden R. Wood, Patcharee Jearanaikoon

**Affiliations:** 1 Biomedical Sciences, Graduate School, Khon Kaen University, Khon Kaen 40002, Thailand; patutongc@kkumail.com; 2 Center for Research and Development of Medical Diagnosis Laboratories, Faculty of Associated Medical Sciences, Khon Kaen University, Khon Kaen 40002, Thailand; moliwo@kku.ac.th (M.W.); patchatip@kku.ac.th (P.T.); 3 Cholangiocarcinoma Research Institute, Khon Kaen University, Khon Kaen 40002, Thailand; 4 Center for Innovation and Standard for Medical Technology and Physical Therapy, Faculty of Associated Medical Sciences, Khon Kaen University, Khon Kaen 40002, Thailand; 5 Center for Biospectroscopy, School of Chemistry, Faculty of Science, Monash University, Monash, VIC 3800, Australia; Kamila.Kochan@monash.edu; 6 Synchrotron Light Research Institute, Nakhon Ratchasima 30000, Thailand; nichada@slri.or.th

**Keywords:** cholangiocarcinoma (CCA), attenuated total reflectance-Fourier transform infrared (ATR-FTIR) spectroscopy, hepatocellular carcinoma (HCC), biliary disease (BD), multivariate analysis, machine learning

## Abstract

**Simple Summary:**

Cholangiocarcinoma is a form of liver cancer that is found, predominantly, in Thailand. Due to the non-specific symptoms and laboratory investigation, it is difficult to rule out cholangiocarcinoma from other liver conditions. Here, we demonstrate the development of a diagnostic tool for cholangiocarcinoma, based on the ATR-FTIR analyses of sera, coupled with multivariate analyses and machine learning tools to obtain a better specificity. The innovative approach that shows highly promising results for this otherwise difficult to diagnose cancer.

**Abstract:**

Cholangiocarcinoma (CCA) is a malignancy of the bile duct epithelium. *Opisthorchis viverrini* infection is a known high-risk factor for CCA and in found, predominantly, in Northeast Thailand. The silent disease development and ineffective diagnosis have led to late-stage detection and reduction in the survival rate. Attenuated total reflectance-Fourier transform infrared spectroscopy (ATR-FTIR) is currently being explored as a diagnostic tool in medicine. In this study, we apply ATR-FTIR to discriminate CCA sera from hepatocellular carcinoma (HCC), biliary disease (BD) and healthy donors using a multivariate analysis. Spectral markers differing from healthy ones are observed in the collagen band at 1284, 1339 and 1035 cm^−1^, the phosphate band ($vsPO_{2}^{-}$) at 1073 cm^−1^, the polysaccharides band at 1152 cm^−1^ and 1747 cm^−1^ of lipid ester carbonyl. A Principal Component Analysis (PCA) shows discrimination between CCA and healthy sera using the 1400–1000 cm^−1^ region and the combined 1800—1700 + 1400–1000 cm^−1^ region. Partial Least Square-Discriminant Analysis (PLS-DA) scores plots in four of five regions investigated, namely, the 1400–1000 cm^−1^, 1800–1000 cm^−1^, 3000–2800 + 1800–1000 cm^−1^ and 1800–1700 + 1400–1000 cm^−1^ regions, show discrimination between sera from CCA and healthy volunteers. It was not possible to separate CCA from HCC and BD by PCA and PLS-DA. CCA spectral modelling is established using the PLS-DA, Support Vector Machine (SVM), Random Forest (RF) and Neural Network (NN). The best model is the NN, which achieved a sensitivity of 80–100% and a specificity between 83 and 100% for CCA, depending on the spectral window used to model the spectra. This study demonstrates the potential of ATR-FTIR spectroscopy and spectral modelling as an additional tool to discriminate CCA from other conditions.

## 1. Introduction

Cholangiocarcinoma (CCA) is a malignancy arising from the bile duct epithelium, which is found, sporadically, all over the world. CCA incidence in western countries was reported between 0.3 and 3.36 per 100,000 people, while in eastern countries, the rate is even higher. The highest incidence was found in Northeast Thailand, which reported 85–118.5 cases per 100,000 people with a high prevalence in Khon Kaen [1,2]. The disease can be caused by various risk factors—primary sclerosing cholangitis, cholelithiasis, biliary disorders, hepatitis B and C infection and lifestyle-related risk, e.g., alcohol consumption and cigarette smoking—, while liver fluke infection (*Opisthorchis viverrini* and *Clonorchis sinensis*) is reported as a common risk of CCA in east Asia [3,4]. Approximately, 10% of chronically infected patients will develop CCA after 30–40 years [2,4].

CCA patients generally have no symptoms, while a long-standing infection and inflammation cause non-specific symptoms, including malaise, jaundice, cholangitis, hepatomegaly, upper quadrant abdominal pain, fatigue, etc. [5]. Unfortunately, a physical examination cannot distinguish CCA from these particular symptoms due to the similarity to other hepatobiliary diseases, especially hepatocellular carcinoma (HCC). Imaging techniques (ultrasound, magnetic resonance imaging (MRI), magnetic resonance cholangiopancreatography (MRCP), computerized tomography (CT) scan) are used to investigate CCA by detecting biliary obstruction, biliary stricture and mass forming. However, these techniques are limited by the cancer itself, as the accuracy depends on the type of tumor, anatomical lesion and tumor size [6]. Laboratory investigations performed by measuring liver function and tumor markers in patient serum are nonspecific for CCA because liver enzymes and bilirubin levels can be elevated in hepatic disorders, while CA19-9 levels can also be found in GI tract cancers [7]. A pathological examination of stained biopsy tissue is the most precise technique and is currently used as a confirmation method. Nevertheless, this technique requires an invasive sample collection, complicated sample handling, time consuming-sample preparation and is labor intensive, which is not suitable for CCA screening or large-scale studies. Potential tumor markers for CCA screening and diagnosis are still intensively investigated in the research process; however, most of these markers require a complicated sample processing and analysis [8]. Although a combination of markers may provide more accurate results [9], the analysis of all markers of interest renders a high cost and is time consuming.

Attenuated Total Reflectance-Fourier Transform Infrared (ATR-FTIR) spectroscopy can be used to detect molecular vibrations of molecules in complex biological samples, including serum [10], which contain a lot of biomolecular information that is useful for a health status assessment. ATR-FTIR spectroscopy has been used to detect cancer-specific biomarkers in serum [11]. Advantages of the ATR-FTIR technique include the ease of sample manipulation and a short measurement time (2 min). Furthermore, ATR-FTIR is a reagent-less technique, requiring only small volumes of a sample that produce a high-signal-to noise ratio output for a further chemometric analysis. Additionally, a single scan of the sample can provide spectral information associated with the molecular phenotype of the disease agent and/or host response [12].

Vibrational spectroscopy, coupled with machine learning algorithms, has previously been applied to sera samples for various diseases, providing an excellent discrimination against controls [13,14]. A study comparing ATR spectra of sera from breast cancer patients versus heathy sera using a Neural Network reported 92–95% sensitivity and 95–100% specificity with the main spectral changes observed in the CH stretching band, C-O from the ribose backbone and P-O vibrations [15]. Toraman et al. [16] applied ATR-FTIR spectroscopy to investigate plasma from colon cancer patients using the multilayer perceptron Neural Network and Support Vector Machine. They reported 76–93% sensitivity, 97–100% specificity using the Neural Network and a 63–90% sensitivity, 80–95% specificity with the SVM [16]. An ATR-FTIR study on sera from patients with brain cancer using SVM reported 93.3% sensitivity and 92.8% specificity [17]. These studies set a precedence for diagnosing other cancers from sera samples with ATR-FTIR spectroscopy.

In our previous study, we reported FTIR spectral discrimination between cholangiocarcinoma and normal tissues and serum samples using an animal model [18]. The discrimination was based on changes in the phosphodiester bands, amino acid, carboxylic ester and collagen molecules in tissue and serum, whereas additional bands corresponding to the amide I, II, polysaccharides and nucleic acid molecules were important in discriminating serum samples from CCA and controls [18]. In this study, we apply ATR-FTIR spectroscopy to investigate human clinical serum samples with the aim to develop a model to discriminate the spectra of CCA from healthy, hepatocellular carcinoma (HCC) and biliary disease (BD) sera using chemometrics. Partial Least Squares Discriminant Analysis (PLS-DA), Support Vector Machine (SVM), Random Forest (RF) and Neural Network (NN) models are established and evaluated by calculating % accuracy, % sensitivity and % specificity.

## 2. Materials and Methods

### 2.1. Human Sera

Sixty samples of CCA, twenty samples of HCC and twenty samples of BD sera were supplied by the Cholangiocarcinoma Research Institute (CARI), Faculty of Medicine, Khon Kaen University, Khon Kaen, Thailand. Fifty healthy sera samples were left over from health checkup program at the Community Medical Laboratory, Faculty of Associated Medical Sciences, Khon Kaen University. Human samples were approved for use by the Center for Ethics in Human Research, Khon Kaen University (HE601117). All sera were aliquoted and kept at −20 °C prior to analyses.

### 2.2. ATR-FTIR Spectroscopy for Serum Analysis

Eight microliters of healthy, CCA, HCC and BD sera was deposited on aluminum foil, air dried and measured using a portable Agilent ATR-FTIR spectrometer 4500 series (Agilent technologies, CA, USA). The parameters for sera measurement were 64 co-added scans for both background and sample, 4 cm^−1^ spectral resolution in the 4000–650 cm^−1^ spectral range with 4 replicates for each sample.

### 2.3. ATR-FTIR Spectral Preprocessing and Analysis

ATR-FTIR spectra acquired from healthy, CCA, HCC and BD human sera were pre-processed by calculating the 2nd derivatives with 15 smoothing points using Savitzky–Golay algorithm and unit vector normalization. Multivariate analysis was performed in 5 spectral ranges: (1) 3000–2800 cm^−1^, (2) 1800–1000 cm^−1^, (3) 1400–1000 cm^−1^ and combine regions, including (4) 1800–1700 + 1400–1000 cm^−1^ and (5) 3000–2800 + 1800–1000 cm^−1^. PCA was performed using The Unscrambler^®^ X (version 10.5, Camo Software, Oslo, Norway). Two-thirds of the samples acquired from each group were categorized as a calibration set to perform supervised analysis, including PLS-DA (The Unscrambler^®^ X version 10.5, Camo Software), Support Vector machine (SVM) (Quasar version 0.9.0, University of Ljubljana, Slovenia), Random Forest (RF) and Neural Network (NN) using multilayer perceptron (Weka software version 3.8.4, The University of Waikato, Hamilton, New Zealand), while averaged spectra from another 1/3 of the samples were appended as a validation set to predict the established model and calculate % accuracy, % sensitivity and % specificity. No technical replicates from the same sample were included in both the training and test set to avoid over optimistic modeling, i.e., the technical replicate trap.

### 2.4. Method Evaluation and Calculation

Predictive results of each model were assigned in Table 1 for comparison of the clinical diagnoses and index test results. Percent accuracy, sensitivity and specificity were calculated by following Formula:

$$\%\;Accuracy=\left(\frac{a+d}{a+b+c+d}\right)\times 100$$

$$\%\;Sensitivity=\left(\frac{a}{a+c}\right)\times 100$$

$$\%\;Specificity=\left(\frac{d}{b+d}\right)\times 100$$

## 3. Results

### 3.1. Characteristic Peaks of Healthy, CCA, HCC and BD Spectra

**Figure 1:**
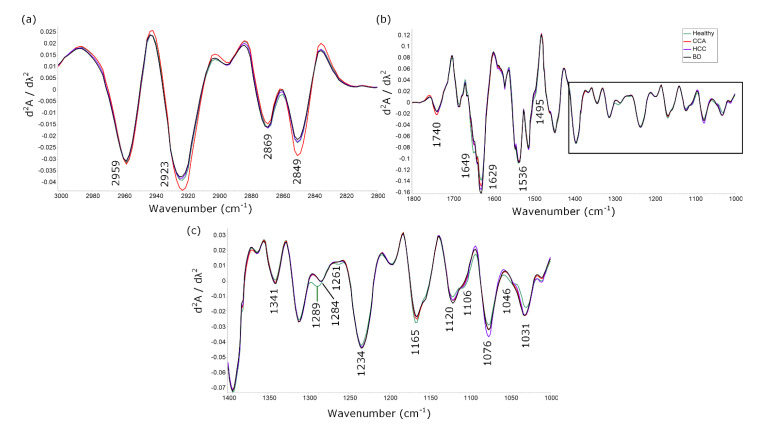
2nd derivative of averaged spectra from healthy (green), CCA (red), HCC (purple) and BD (black) sera in (**a**) CH stretching region (3000–2800 cm^−1^), (**b**) fingerprint spectral (1800–1000 cm^−1^) and (**c**) 1400–1000 cm^−1^ region.

Averaged 2nd derivative spectra of healthy, CCA, HCC and BD sera from the CH stretching region (3000–2800 cm^−1^) and fingerprint spectral region (1800–1000 cm^−1^) are shown in Figure 1a,b, respectively. A spectral shift from 1289 cm^−1^ in the healthy group to 1284 cm^−1^ in CCA, HCC and BD was observed, which indicated an alteration of collagen molecules. A shoulder at ~1106 and ~1046 cm^−1^ was found in the healthy group assigned to the C-O stretching and bending vibration in the C-OH group of carbohydrate (Figure 1c) [19].

However, a clear spectral difference could not be observed in the CH stretching region and amide I, II region. The averaged spectra in certain regions may not provide informative data due to their common functional molecule or the abundant protein found in serum; hence, chemometric approaches were required to further interrogate the data.

### 3.2. CCA Spectral Discrimination Using Unsupervised Analysis: Principal Component Analysis (PCA)

**Figure 2:**
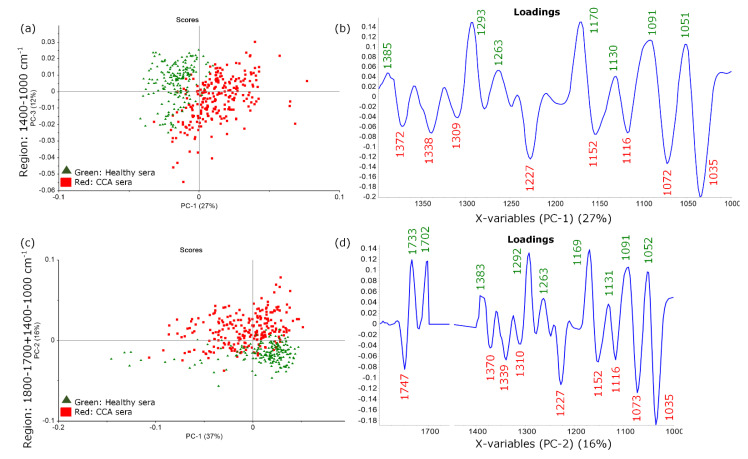
PCA results of healthy (green) and CCA (red) sera displayed in (**a**) PC-1,3 scores plot in the 1400–1000 cm^−1^ spectral region, (**b**) corresponding PC-1 loadings plot and (**c**) PC-1,2 scores plot using the combined 1800–1700 + 1400–1000 cm^−1^ spectral regions and (**d**) corresponding PC-2 loadings plot.

Spectra acquired from 60 CCA and 50 healthy serum samples were preprocessed and analyzed using the Principal Component Analysis (PCA). The PCA scores plot for the 1400–1000 cm^−1^ region showed discrimination along PC1 (Figure 2a), while the combined regions 1800–1700 + 1400–1000 cm^−1^ showed discrimination along PC2 (Figure 2c). The loadings plot for the 1400–1000 cm^−1^ region (Figure 2b) showed wavenumber values at 1372, 1338, 1309, 1227, 1152, 1116, 1072 and 1035 cm^−1^ corresponding to the CCA sera. The additional wavenumber value at 1747 cm^−1^ of CCA sera was observed when using the combined region (1747, 1370, 1339, 1310, 1227, 1152, 1116, 1073 and 1035 cm^−1^) (Figure 2d). Peaks at ~1072 and ~1116 cm^−1^ were assigned to P-O-C modes from phosphodiester groups in nucleic acids. The discrimination was also based on variance in the amide III region that included contributions from collagen (~1400–1200 cm^−1^ and 1035 cm^−1^) derived from CH_2_ bending and in-plane of NH bending and CN stretching vibrations of the protein and CH_2_ of collagen [20,21]. Bands at 1152 cm^−1^ along with ~1073 cm^−1^ can be indicated as glycogen or polysaccharide molecules [22]. The ester carbonyl band in CCA appeared at 1747 cm^−1^, while, in uninfected patients, it shifted to 1733 cm^−1^. A band at 1702 cm^−1^ was assigned to the C=O vibration of nucleic bases [23].

A PCA analysis was also performed in the CH stretching region (3000–2800 cm^−1^), fingerprint spectral region (1800–1000 cm^−1^) and a combination of the 3000–2800 + 1800–1000 cm^−1^ spectral regions. However, the PCA scores plots showed no discrimination among the two groups of sera (Figure S1). Furthermore, the spectra of 20 HCC, 20 BD and 60 CCA sera were evaluated with a PCA analysis. The PCA scores plots showed no discrimination among HCC vs. CCA and BD vs. CCA sera (Figure S2). A supervised analysis using the Partial Least Squares Discriminant Analysis (PLS-DA) was then applied to provide a better discrimination and establish the PLS-DA calibration model for CCA sera.

### 3.3. Establishment and Evaluation of CCA Predictive Model Using Partial Least Squares Discriminant Analysis (PLS-DA)

**Figure 3:**
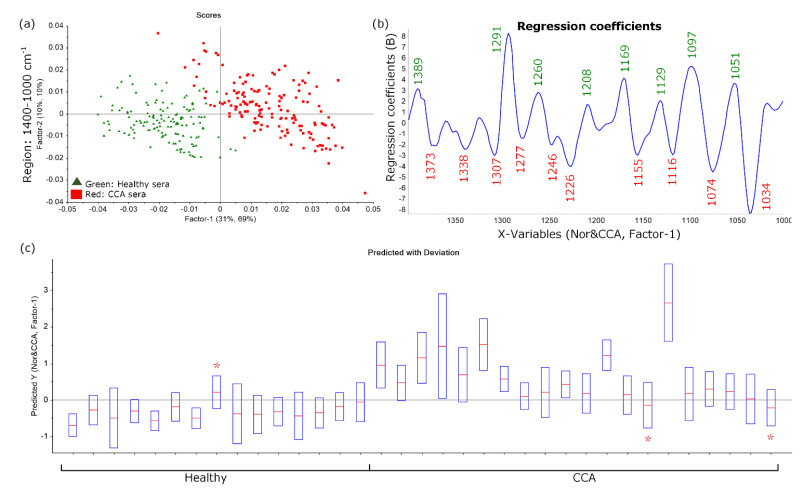
PLS-DA results from ATR-FTIR sera spectra, healthy (green) and CCA (red) display in (**a**) scores plots, (**b**) regression coefficients and (**c**) predictive result at 1400–1000 cm^−1^. The stars (*) indicate the false prediction samples in the model which give 1 false positive and 2 false negative predictions.

Spectra were categorized into two groups: a calibration set and a prediction set. Forty of the CCA and thirty-five of the healthy sera were modeled in the calibration set and analyzed using PLS-DA to generate a PLS predictive model. The averaged spectra of the remaining samples (20 CCA and 15 healthy) were predicted using the generated PLS model for various spectral regions. The sensitivity and specificity for each of the spectral regions are shown in Table 2. The PLS model in the fingerprint spectral region (1800–1000 cm^−1^) showed discrimination along PC1 (*x*-axis) (Figure S3a). The regression coefficients (Figure S3b) showed wavenumber values from the 1743 cm^−1^ C=O lipid ester carbonyl, 1687, 1665, 1630 and 1555 cm^−1^ from C=O and N-H vibrational modes of proteins, 1512 cm^−1^ of N-H or C-N vibrations and the combination of polysaccharide, glycogen, amide III, collagen and phosphodiester modes from nucleic acids at lower wavenumber values (1450, 1408, 1371, 1337, 1307, 1277, 1246, 1225, 1154, 1117, 1074 and 1034 cm^−1^) corresponding to CCA sera samples. The PLS model in 1400–1000 cm^−1^ spectral region showed a clear discrimination along Factor-1 (*x*-axis) (Figure 3a). The regression coefficients plot (Figure 3b) appeared to have a similar profile to PLS-DA performed on the 1800–1000 cm^−1^ region. Moreover, the discrimination trend could also be found in the combined region of 1800–1700 + 1400–1000 cm^−1^ (Figure S3c) and 3000–2800 + 1800–1000 cm^−1^ (Figure S3e), while the CH stretching region alone (3000–2800 cm^−1^) showed no discrimination between the two groups (Figure S3d).

According to the predictive model, the positive values were predicted as CCA, while the negative values were predicted as healthy. The modelling performed in five spectral regions, ranging from 62 to 91% accuracy, 70 to 90% sensitivity and 53 to 93% specificity. The results showed that the 1400–1000 cm^−1^ spectral region (Figure 3c) provided the best prediction with 14 healthy and 18 CCA, giving one false positive and two false negatives, based on the minimizing of major proteins, e.g., albumin and globulin in the amide I and II region. This indicated that the PLS-DA provided a better discrimination between healthy and CCA sera compared to the unsupervised analysis (PCA).

We further attempted to differentiate between different disease patient groups, which developed similar clinical symptoms and laboratory test results and, hence, difficult for physicians to diagnose. PLS-DA was performed on CCA vs. HCC and CCA vs. BD samples in five spectral regions. Figure S4 shows the PLS scores plots of CCA vs. HCC and CCA vs. BD, the results indicated no discrimination among each group so a more advanced machine modelling was required to achieve the differentiation among disease groups.

### 3.4. Advanced Machine Modelling of CCA Serum

A more advanced machine learning was performed using a Support Vector Machine (SVM), Random Forest (RF) and Neural Network (NN). The models were established in five spectral ranges using vector normalized 2nd derivative spectra, 2/3 of the dataset was used as the calibration set and 1/3 used as the validation set. Firstly, SVM was applied as a nonlinear analyzing tool for spectral data, which contained high dimensional input attributes. A radial basis function kernel was chosen for the SVM learning. The 1400–1000 cm^−1^ spectral model gave the best predictive values for a differentiation of CCA sera from healthy sera with a 94% accuracy, 95% sensitivity and 93% specificity, and from HCC patients with a 85% accuracy, 100% sensitivity and 33% specificity. For a differentiation of CCA from BD, the best prediction was obtained from three spectral regions—1800–1000 cm^−1^, 1800–1700 + 1400–1000 cm^−1^ and 3000–2800 + 1800–1000 cm^−1^—, with equal accuracy, sensitivity and specificity at 77%, 90% and 33% (Table 2). Moreover, candidate scatter plots of 5 spectral ranges were showed in Table S1. Although SVM had an improved better sensitivity to discriminate CCA from other groups, the specificity was limited.

To obtain a better specificity, other learning algorithms were applied to analyze these spectral data. The analysis using RF was performed with a bagging learner, 100 iterations and 100 batch sizes using a 10-fold cross-validation. The best predictive values for a differentiation of CCA from healthy and HCC obtained by using the combined regions, 3000–2800 + 1800–1000 cm^−1^, resulted in an equal 100% sensitivity with 93% and 33% specificity, respectively. For the CCA and BD model, the 3000–2800 cm^−1^ spectral region was found to be the best model for a differentiation with 95% sensitivity and 33% specificity. Thus, RF was still limited in specificity.

The NN analysis was finally performed by multilayer perceptron with one hidden layer, which varied the number of nodes from 0 to 35 nodes and one default parameter to identify the network which provided the best sensitivity, specificity and accuracy. Each model was set with the same parameters: 0.3 learning rate, 0.2 momentum and 500 epochs in a 10-fold cross-validation. Compared with the other advance model, NN improved the prediction outcome in CCA and the healthy model up to a 100% accuracy, 100% sensitivity and 100% specificity at the combined spectral region at 3000–2800 + 1800–1000 cm^−1^; however, the CH stretching region (3000–2800 cm^−1^) alone resulted in the worst values. This combined spectral region with no hidden layer tended to be the best model to differentiate CCA from healthy sera samples (input: hidden node: output = 541: 0: 2) (Table S2).

For the CCA and HCC models, the 100% sensitivity was obtained at the 1400–1000 cm^−1^ and the combined spectral regions, but with a rather low specificity. The best compromised model at 1800–1000 cm^−1^ (input: hidden node: output = 432: 2: 2) was suggested with a 92% accuracy, 95% sensitivity and 83% specificity. In the CCA and BD model, the spectral regions 3000–2800 + 1800–1000 cm^−1^ gave the highest accuracy, sensitivity and specificity with 81%, 80% and 83%, respectively (input:hidden node:output = 541:14:2).

## 4. Discussion

In our previous study [18], we reported the discrimination of *O. viverrini* + NDMA infected from uninfected hamster sera using PCA in the fingerprint spectral region (1800–900 cm^−1^). The important spectral signatures included: (i) a band at 1745 cm^−1^ assigned to the lipid ester carbonyl C=O, (ii) bands at 1380–1200 cm^−1^ and 1034 cm^−1^ from collagen, iii) a band at 1071 cm^−1^ from nucleic acid phosphodiester groups and iv) a band at 1153 cm^−1^ from polysaccharide molecules (Table 3). These bands were also observed in the current study and compared with the animal study in Table 3.

The band at ~1074 cm^−1^ observed in serum was tentatively assigned to circulating tumor DNA (ctDNA) fragments that were characteristic of cancer and were released into the blood stream [11,24] or, alternatively, from phosphorylated proteins, which were also found in the serum [25]. The observed changes in the carbohydrate region 1300–1000 cm^−1^ could be explained by two phenomena: the changes in the sugar backbone of nucleic acids and an elevation in carbohydrates [26] and the byproduct of glucose consumption or glycogen degradation related to cancer metabolism and cancer progression [27,28,29]. Furthermore, collagen released from the tumor microenvironment could be indicative of cancer progression and, thus, be a prognostic indicator for cancer [30,31]. Prakobwong et al. reported a significantly increase in type I collagen, hydroxyproline (HYP), metrixmetalloproteinase-7 (MMP-7) and the tissue inhibitor of MMP-2 (TIMP-2) levels in CCA plasma compared to healthy and benign biliary disease [32]. The specific protein spectral signatures in sera could be derived from the discharge of some protein from the tumor microenvironment [33]. Additionally, the tumor markers, which were found in the circulating system, could be CA19-9, α1β-glycoprotein, afamin, MMP-7, HYP, collagen I and γ-Glutamyltransferase, etc. [8].

The infrared spectral signatures corresponding to CCA presented in this study were obtained from the comparison of CCA and healthy sera. However, these spectral peaks can be observed in various diseases, e.g., the study of cirrhosis and HCC compared to healthy sera by Thumanu et al. [43]. The abnormalities in liver can be observed in sera spectra via an alteration of a protein secondary structure and higher lipid accumulation. They reported a decrease in α-helix at 1648 cm^−1^ and an increase in the β-sheet secondary structure of protein at 1639 cm^−1^ in HCC and cirrhosis. Additionally, the peak at ~1745 cm^−1^ was found in cirrhosis sera which confirmed a state of a high lipid level. Sitnikova et al. [44] also reported the significance differences between healthy and breast cancer sera in the spectral range 1306–1250 cm^−1^ corresponding to the vibrations of several functional groups of DNA and RNA. Our results also agreed with a recent study on sera from brain cancer patients using ATR spectroscopy, with changes in the 1540–1490 cm^−1^ and ~1036 cm^−1^ regions being observed [45]. Due to the similar biochemical components associated with CCA, HCC and BD sera being closely related with each other [46], the infrared spectra also showed similar profiles and, therefore, the discrimination specificity was decreased among three groups. To enable a more robust classifier, sophisticated mathematical and chemometric methods were necessary to identify discriminating zones reflecting a different molecular composition of the spectra [47]. Consequently, more advance machine modelling was required to achieve a higher degree of differentiation.

According to the diagnostic limitation in CCA, the exact sensitivity and specificity of each diagnostic technique could not be easily determined. Thus, the integrated techniques are often used to provide a better diagnostic efficacy [48,49,50,51]. The tumor marker, CA 19-9, showed a 50–100 % sensitivity and 50–98% specificity in PSC patients, whereas a combination with imaging techniques showed a 78% sensitivity and 67% specificity [52,53]. The elevation of CEA levels, which is an indicator of glycoprotein produced by the GI tract, reported a 33–84% sensitivity and 33–100% specificity, while the combination of CA 19–9 and CEA levels provided a 86% accuracy in a PSC patient [53]. Several studies have reported several efficient biomarkers for CCA diagnosis. However, the monitoring of these markers requires special equipment along with complicate analysis procedures [8,54]. Meanwhile, our candidate CCA spectral modelling using PLS-DA, SVM, RF and NN resulted in a 90% sensitivity and 93% specificity, 90–100% sensitivity and 33–93% specificity, 95–100% sensitivity and 33–93% specificity and 80–100% sensitivity and 83–100% specificity, respectively.

Several studies on the application of FTIR spectroscopy and chemometric analysis for cancer diagnosis using sera have been reported. For example, an ATR-FTIR study on brain cancer sera using SVM provided a high sensitivity and specificity with 93.3% and 92.8%, respectively [17]. Another study evaluated the diagnostic model of brain cancer using Random Forest, PLS-DA and SVM, which provided sensitivities and specificities of 93.8% and 80.1%, 95.9% and 81.7% and 92.1% and 88.7%, respectively [45]. A study of breast cancer performed using PCA-LDA reported a 92% sensitivity, 85% specificity and 90% accuracy, while a cluster analysis gave a 96% sensitivity, 93% specificity in the 1250–685 cm^−1^ region [21]. Another breast cancer study reported a Neural Network modelling efficacy with a 92–95% sensitivity and 95–100% specificity [15]. The differentiation accuracy, test sensitivity or specificity did not only depend on the learning algorithm, but also the input attributes, complexity of data and sample size. Hitherto, there is no consensus on the best analysis method.

This study demonstrated the advantage of ATR-FTIR coupled with a multivariate analysis or advance machine modelling to differentiate the CCA and other similar conditions and, thus, showed a promising additional tool for cancer identification. The differentiation between disease (CCA) and healthy sera was more effective than among similar disease conditions (HCC and BD). CCA-specific analytes could be found in serum, which is the most complex biofluid carrying over 20,000 different proteins [11]. The FTIR spectroscopic technique was suitable for the multi-molecular biochemical analysis, where a single spectrum can provide biomolecular information on multiple analytes [12]. However, differentiation among diseases can reduce the % specificity of the model and the judicious selection of spectral regions was required to improve the specificity.

## 5. Conclusions

The study herein demonstrated the potential of ATR-FTIR spectroscopy as a clinical tool. A spectral shift from 1289 cm^−1^ in the healthy group to 1284 cm^−1^ in CCA, HCC and BD was observed in 2nd derivative spectra, which indicated changes of collagen molecules in serum. Subsequently, spectral bands of collagen were confirmed at ~1339 and 1035 cm^−1^ from both PCA and PLS-DA analyses. Moreover, ~1073 cm^−1^ of phosphate groups, ~1747 cm^−1^ of lipid ester carbonyl and ~1152 cm^−1^ of polysaccharides were also observed in loading plots of CCA sera in both human and hamster. A spectral analysis using a multivariate analysis and machine learning tools revealed the differentiation efficacy of CCA from other underlying diseased and healthy sera. The NN provided the best sensitivity and specificity of CCA with 80–100% and 83–100% in the selected spectral model. The portable, simple procedure, rapid and robust features of ATR-FTIR make it an ideal screening technique for a serum analysis. To test the true efficacy of the approach, a large-scale clinical trial is required to assess the true specificity against other cancers.

## Figures and Tables

**Table 1:** Table defines the prediction performance between reference and index tests.

Index Test	Clinical Diagnoses	
(Predictive Model)	CCA	Other Condition
CCA	*a*	*b*
Other condition	*c*	*d*

**Table 2:** Evaluation of CCA predictive models in different spectral regions.

Models		Spectral Range														
		3000–2800 cm^−1^			1800–1000 cm^−1^			1400–1000 cm^−1^			1800–1700 + 1400–1000 cm^−1^			3000–2800 + 1800–1000 cm^−1^		
		%Acc	%Sen	%Spec	%Acc	%Sen	%Spec	%Acc	%Sen	%Spec	%Acc	%Sen	%Spec	%Acc	%Sen	%Spec
PLS-DA	Healthy/CCA	62	70	53	80	90	67	**91**	**90**	**93**	83	90	73	80	90	67
SVM	Healthy/CCA	86	85	87	94	95	93	**94**	**95**	**93**	94	95	93	94	95	93
	CCA/HCC	73	95	0	81	100	17	**85**	**100**	**33**	81	100	17	81	100	17
	CCA/BD	73	95	0	**77**	**90**	**33**	73	85	33	**77**	**90**	**33**	**77**	**90**	**33**
RF	Healthy/CCA	71	85	53	97	100	93	94	100	87	94	100	87	**97**	**100**	**93**
	CCA/HCC	73	95	0	81	100	17	81	95	33	77	90	33	**85**	**100**	**33**
	CCA/BD	**81**	**95**	**33**	73	85	33	77	90	33	77	90	33	77	90	33
NN	Healthy/CCA	82	90	73	97	95	100	97	95	100	97	95	100	**100**	**100**	**100**
	CCA/HCC	84	95	50	**92**	**95**	**83**	92	100	67	88	100	50	88	100	50
	CCA/BD	80	85	66	81	80	33	73	70	83	81	85	67	**81**	**80**	**83**

Definitions: %Acc—% accuracy; %Sen—% sensitivity; %Spec—% specificity; PLS-DA—Partial Least Square Discriminant Analysis; SVM—Support Vector Machine; RF—Random Forest; NN—Neural Network. Bold words indicate the best predictive values in each model.

**Table 3:** FTIR spectral markers important in the discrimination of CCA from healthy human serum and hamster serum using PCA and PLS-DA.

Biomolecule	Molecular Vibration	Wavenumber (cm^−1^)				References
		PCA		PLS-DA		
		Human Serum	Hamster Serum	Human Serum	Hamster Serum	
Lipid	C=O	1747	1745	1743	1736	[34,35]
Collagen	Amide III and CH_2_ wagging	1380–1200	1380–1200	1380–1200	1380–1200	[36,37,38,39,40]
	CH_2_ vibration	1339	~1337	1337	~1337	
		1035	1030	1034	1030	[41]
Nucleic acid or Protein	PvsO2−	1073	1077	1074	1076	[23,25,34,42]
Polysaccharide	C-O stretching	1152	1156	1154	1153	[22,23]

## Data Availability

The data presented in this study are available in this article (and Supplementary Materials).

## Supplementary Materials

The following are available online at https://www.mdpi.com/article/10.3390/cancers13205109/s1, Figure S1: PCA scores plots of healthy and CCA sera using CH stretching region, fingerprint spectral region and the combined spectral region (3000–2800 + 1800–1000 cm^−1^), Figure S2: PCA scores plots of CCA vs. HCC and BD sera using CH stretching region, fingerprint spectral region and 1400–1000 cm^−1^, Figure S3: PLS-DA scatter plots of healthy vs. CCA sera in fingerprint spectral region and corresponding regression coefficients for the fingerprint spectral region and the scores plots at CH stretching region and combine region 1800–1700 + 1400–1000 cm^−1^, 3000–2800 + 1800–1000 cm^−1^ spectral windows, Figure S4: PLS-DA scores plots of CCA vs. HCC and BD sera using CH stretching region, fingerprint spectral region and 1400–1000 cm^−1^, Table S1: indicated Support Vector Machine candidate scatter plots of CCA versus healthy, HCC and BD in the 5 spectral regions, Table S2: number of nodes in 1 defined as the hidden layer which gave the best % accuracy, % sensitivity and % specificity in each spectral range.
