# Supplementary material for: Detection of Human Cholangiocarcinoma Markers in Serum Using Infrared Spectroscopy
Source: Cancers (Basel). 2021 Oct 12;13(20):5109. doi: 10.3390/cancers13205109 (PMC8534168; doi:10.3390/cancers13205109)
Supplement: Supplementary file 1 [file cancers-13-05109-s001.zip › cancers-1403850-supplementary.pdf]

Supplementary Materials: Detection of Human Cholangiocarcinoma Markers in Serum using Infrared Spectroscopy

Patutong Chatchawal, Molin Wongwattanakul, Patcharaporn Tippayawat, Kamilla Kochan, Nichada Jearanaikoon, Bayden R. Wood and Patcharee Jearanaikoon

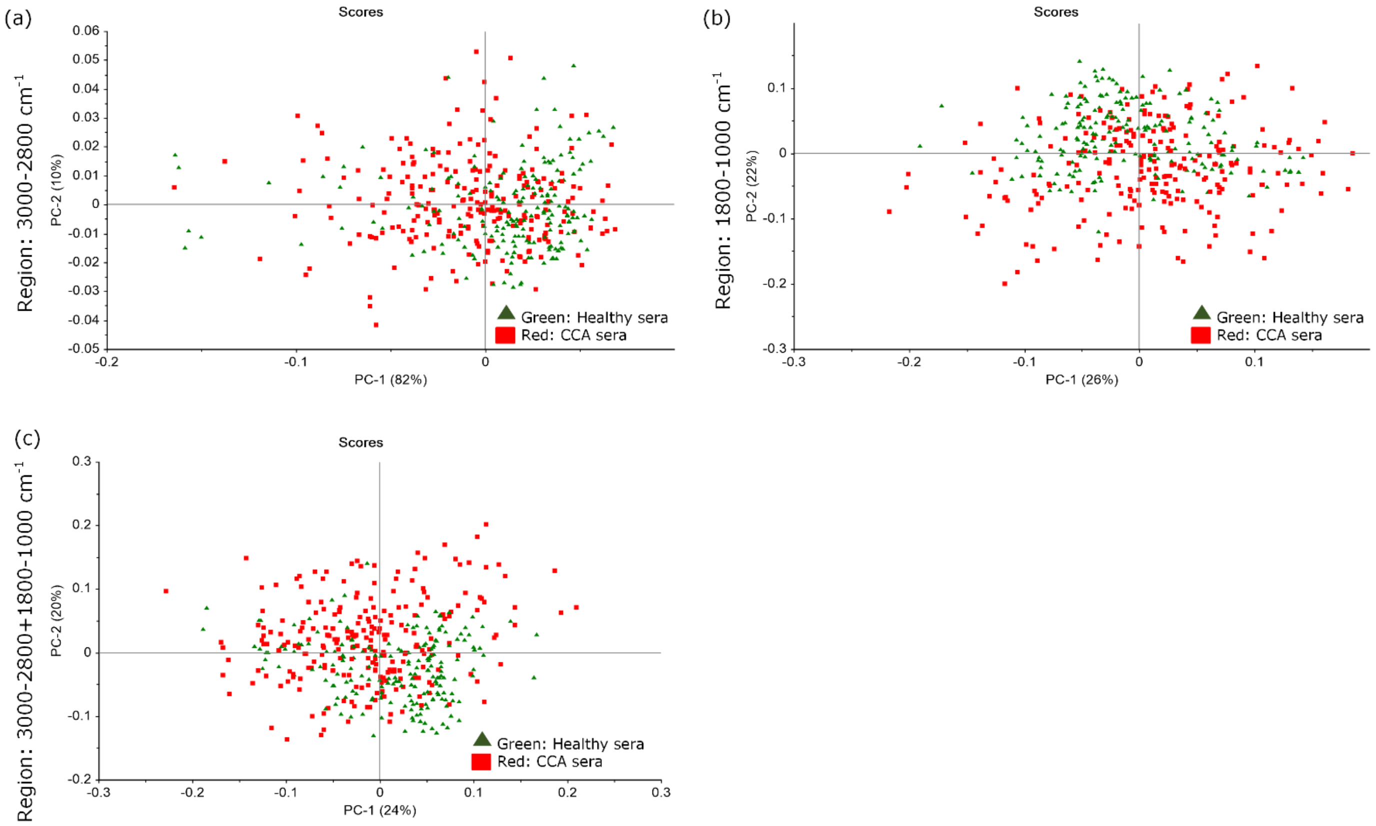

Figure S1 PCA scores plots of healthy (green) and CCA (red) sera using: (a) CH stretching region (3000–2800 cm^{-1}), (b) fingerprint spectral region (1800–1000 cm^{-1}) and the combined spectral region including the CH stretching and fingerprint spectral windows. The scores plots show no discrimination for both groups of sera.

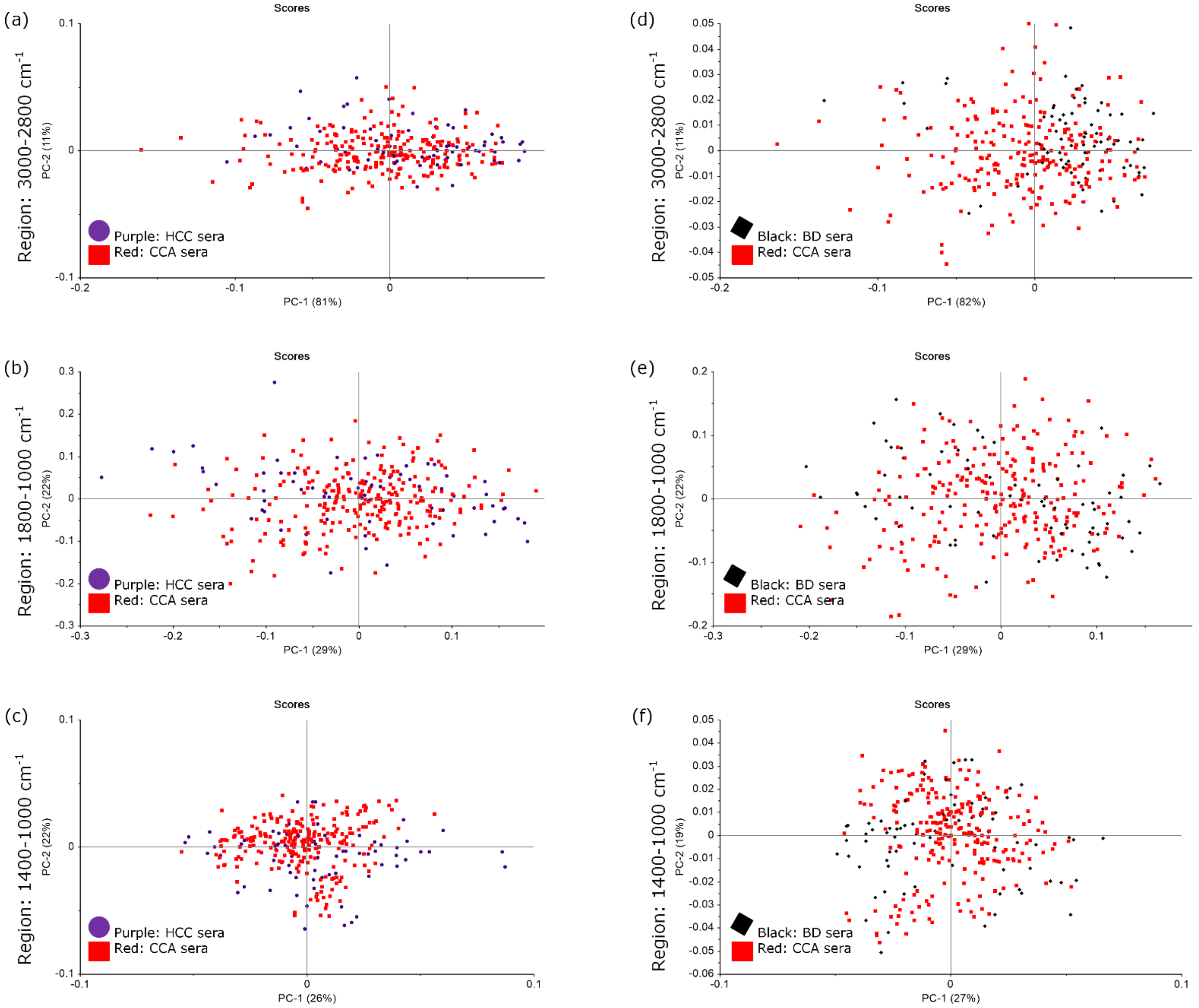

Figure S2 PCA scores plots of CCA (red) and HCC (purple) sera using: (a) CH stretching region (3000–2800 cm^{-1}), (b) fingerprint spectral region (1800–1000 cm^{-1}) and (c) 1400–1000 cm^{-1}, which shows no discriminations along PC1 and PC2. There is also no discrimination observed in the PCA scores plots of CCA (red) and BD (black) sera in (d) CH stretching region (3000–2800 cm^{-1}), (e) fingerprint spectral region (1800–1000 cm^{-1}) and (f) 1400–1000 cm^{-1}. The analysis was also performed in other 2 combined regions and the result showed no discrimination among 2 groups (data not show).

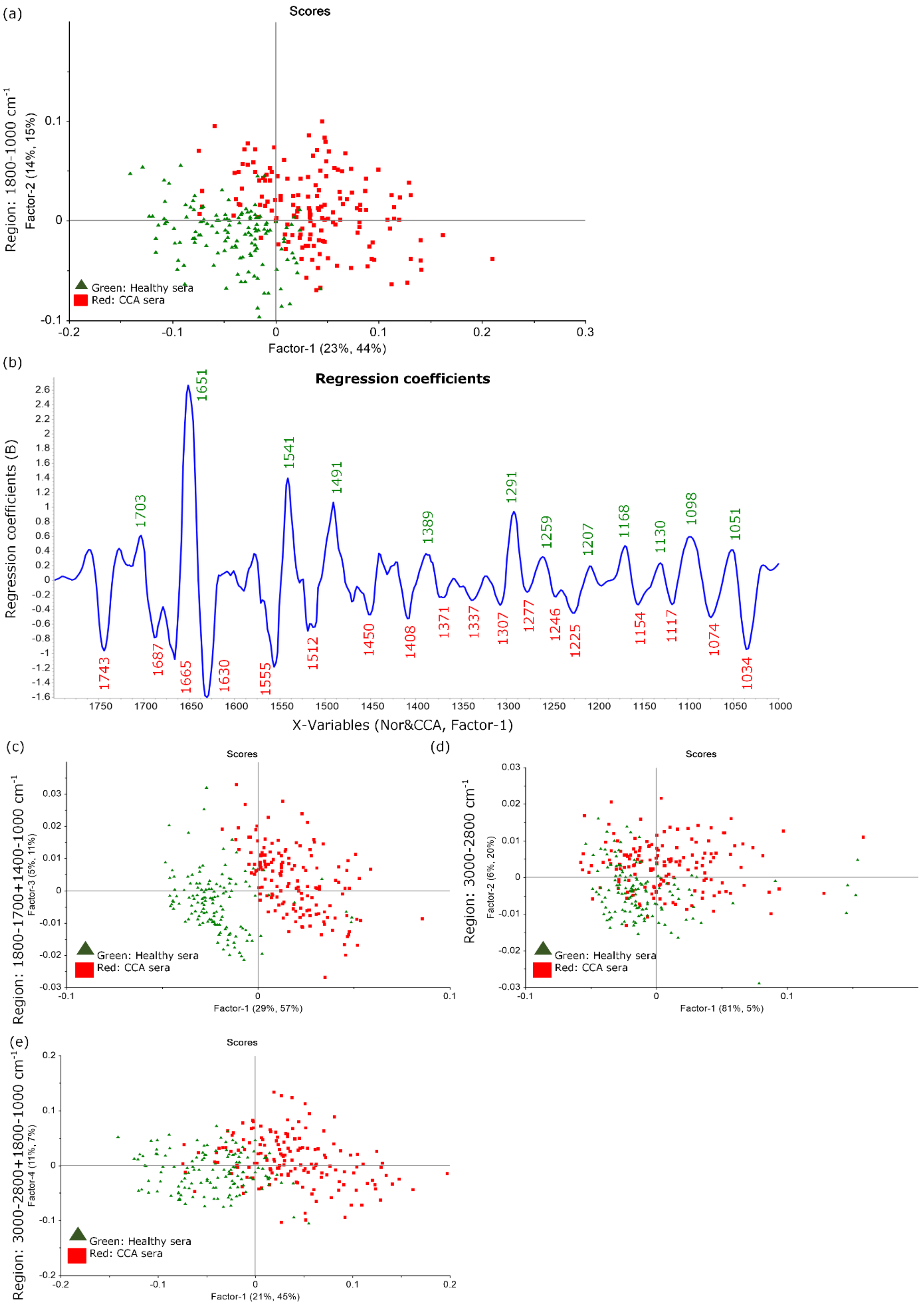

Figure S3 PLS-DA scatter plots of healthy (green) vs CCA (red) sera in: (a) fingerprint spectral region (1800–1000 cm^{-1}) and corresponding regression coefficients for the fingerprint spectral region shown in (b), (c) combine region at 1800–1700+1400–1000 cm^{-1}, (d) CH stretching region (3000–2800 cm^{-1}) and (e) the combined regions including the 3000–2800+1800–1000 cm^{-1} spectral windows.

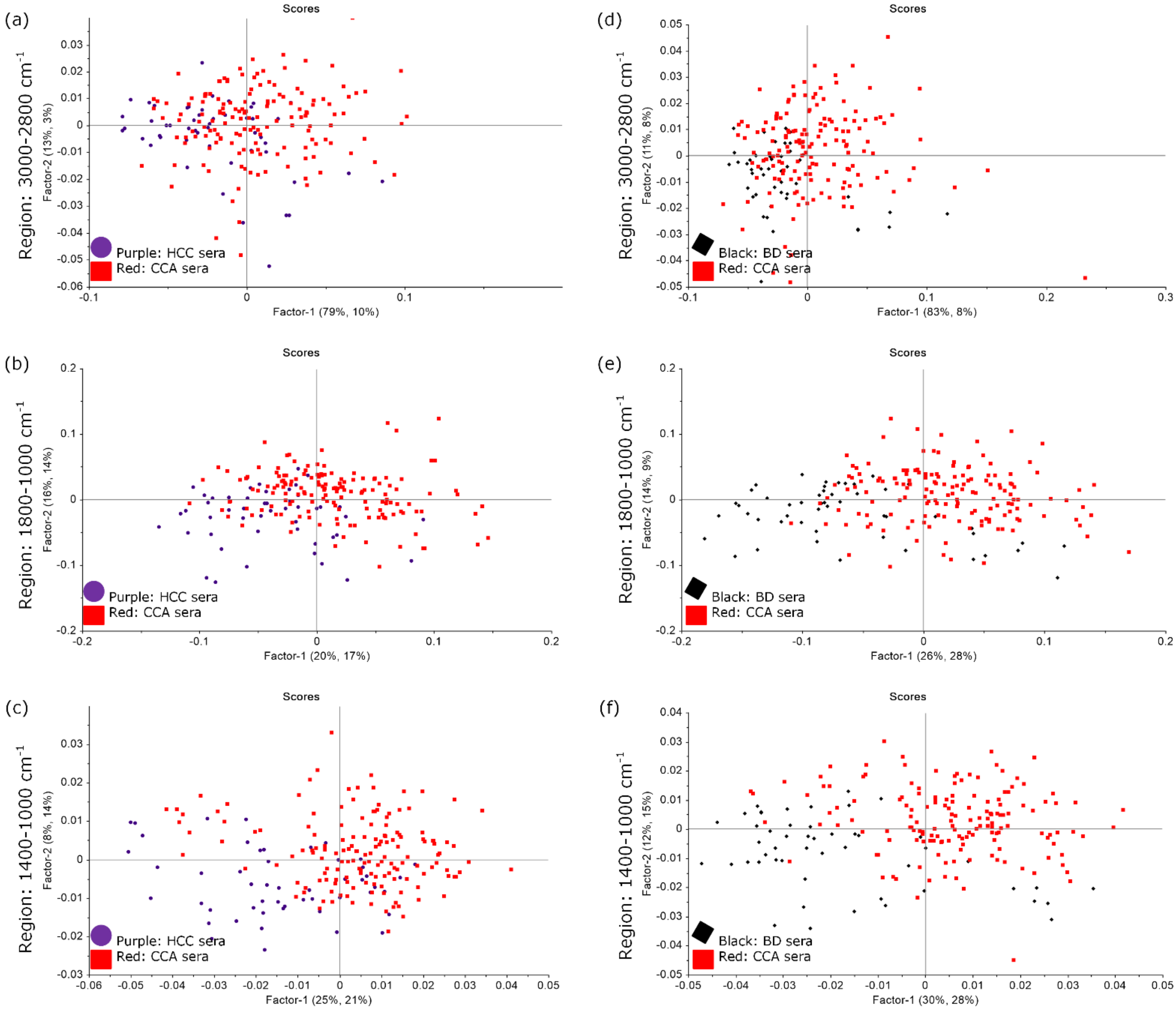

Figure S4 PLS-DA scores plots show no discrimination among CCA (red) vs HCC (purple) sera and CCA (red) and BD (black) sera in (a, d) CH stretching region (3000–2800 cm^{-1}), (b, e) fingerprint spectral region (1800–1000 cm^{-1}) and (c, f) 1400–1000 cm^{-1}, respectively. The analysis was also performed in other 2 combined regions and the result showed no discrimination among 2 groups (data not shown).

Table S1. Indicate support vector machine candidate scatter plots of CCA versus healthy, HCC and BD in the 5 spectral regions.

Spectral region	CCA vs Healthy	CCA vs HCC	CCA vs BD
3000–2800 cm^{-1}	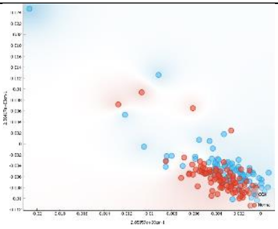<br>2858/2864	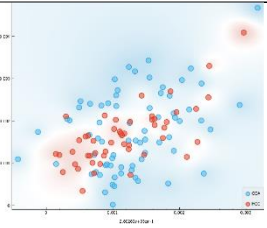<br>2802/2810	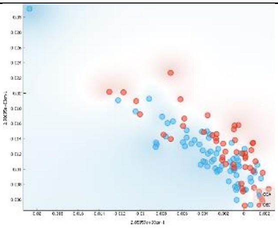<br>2858/2906
1800–1000 cm^{-1}	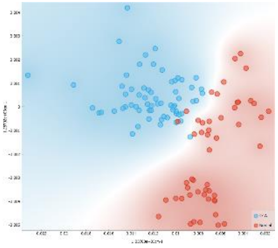<br>1037/1287	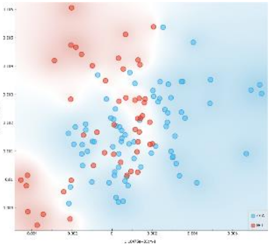<br>1104/1270	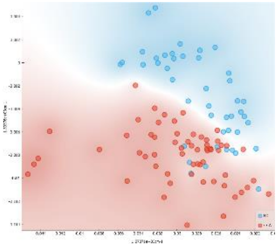<br>1078/1303
1400–1000 cm^{-1}	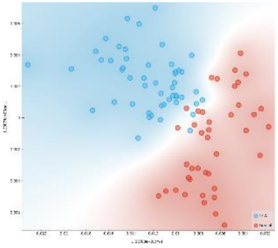<br>1037/1290	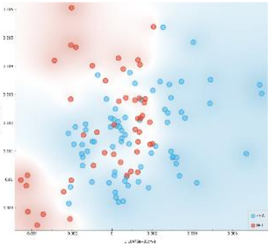<br>1104/1270	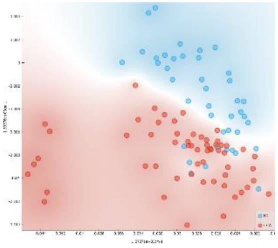<br>1078/1303
1800–1700+<br>1400–1000 cm^{-1}	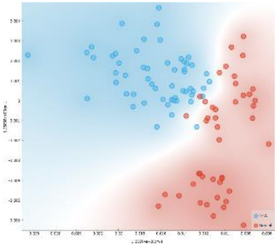<br>1035/1288	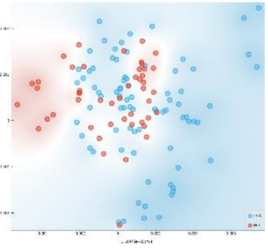<br>1104/1800	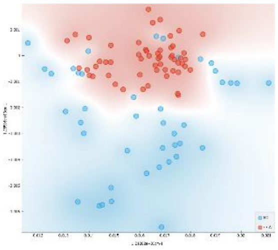<br>1212/1285
3000–2800+<br>1800–1000 cm^{-1}	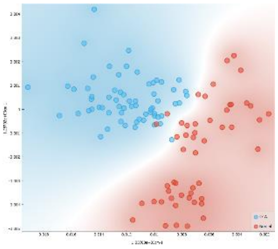<br>1037/1287	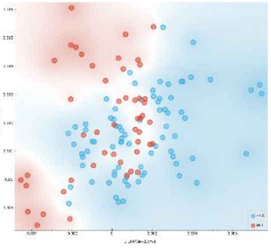<br>1104/1270	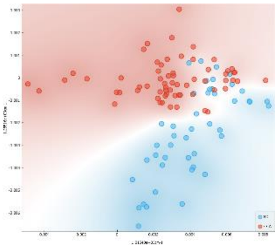<br>1015/1285

Table S2. Number of nodes in 1 defined as the hidden layer which gave the best %accuracy, %sensitivity and %specificity in each spectral range.

Spectral region (cm^{-1})	Number of input (nodes)	Number of nodes in 1 hidden layer		
		CCA vs Healthy	CCA vs HCC	CCA vs BD
3000–2800	110	2, 5, 15, 19	2	3
1800–1000	432	0–35, a(217)	2, 18, 21, 23, 27, 28, 31–35, a(217)	8
1400–1000	217	0–35, a(110)	2, 15, 20–22, 29–35, a(110)	13, 20
1800–1700+1400–1000	271	0–35, a(137)	0, 2, 3, 5, 16, 25, 27, 28, 32, 33, 35	0
3000–2800+1800–1000	541	0, 1, 3-5, 8–22, 24–35, a(272)	2, 4, 6–8, 13, 15–26, 31-35	14, 15, 21

"a" defines a default parameter from the program which equal to the mean value of input attributes and output classes
